# *Wfs1*^*E864K*^ knock-in mice illuminate the fundamental role of Wfs1 in endocochlear potential production

**DOI:** 10.1038/s41419-023-05912-y

**Published:** 2023-06-29

**Authors:** Elodie M. Richard, Emilie Brun, Julia Korchagina, Lucie Crouzier, Corentin Affortit, Stacy Alves, Chantal Cazevieille, Anne-Laure Mausset-Bonnefont, Marc Lenoir, Jean-Luc Puel, Tangui Maurice, Marc Thiry, Jing Wang, Benjamin Delprat

**Affiliations:** 1 MMDN, Univ Montpellier, EPHE, INSERM, Montpellier, France; 2 INM, Univ Montpellier, INSERM, Montpellier, France; 3 IRMB, Univ Montpellier, INSERM, Montpellier, France; 4 Laboratoire de Biologie Cellulaire, Université de Liège, Liège, Belgique

**Keywords:** Neurodegenerative diseases, Cochlea

## Abstract

Wolfram syndrome (WS) is a rare neurodegenerative disorder encompassing diabetes *mellitus*, diabetes *insipidus*, optic atrophy, hearing loss (HL) as well as neurological disorders. None of the animal models of the pathology are presenting with an early onset HL, impeding the understanding of the role of Wolframin (WFS1), the protein responsible for WS, in the auditory pathway. We generated a knock-in mouse, the *Wfs1*^*E864K*^ line, presenting a human mutation leading to severe deafness in affected individuals. The homozygous mice showed a profound post-natal HL and vestibular syndrome, a collapse of the endocochlear potential (EP) and a devastating alteration of the stria vascularis and neurosensory epithelium. The mutant protein prevented the localization to the cell surface of the Na^+^/K^+^ATPase β1 subunit, a key protein for the maintenance of the EP. Overall, our data support a key role of WFS1 in the maintenance of the EP and the stria vascularis, via its binding partner, the Na^+^/K^+^ATPase β1 subunit.

## Introduction

Wolfram syndrome (WS) is an emblematic form of inherited optic neuropathies defined by progressive and rapid vision loss caused by optic atrophy (OA), sensorineural hearing loss (HL), and various symptoms such as cerebellar ataxia and peripheral neuropathy. In addition to this wide neurosensory impairment, affected individuals develop an early onset diabetes *mellitus* often associated with diabetes *insipidus* and hypogonadism [1]. WS is caused by variants in *WFS1* gene, inherited in an autosomal recessive fashion. Interestingly, there is a huge variability in symptom presentation and severity. If the majority of the affected individuals present OA and diabetes *mellitus*, only 60% of them develop deafness [1]. Variants in *WFS1* have also been associated with several other forms of deafness. Dominant variants of *WFS1* may lead to non-syndromic low-frequency sensorineural HL [2, 3], syndromic profound HL associated with OA and glucose intolerance [4, 5] and a novel congenital disorder encompassing neonatal/early-onset diabetes, congenital cataracts, and sensorineural HL [6]. All these entities with the clinical triad of congenital progressive HL, diabetes *mellitus*, and OA are commonly denominated as Wolfram-like syndrome diseases [7]. In addition, it has been shown that recessive mutations in *WFS1* also cause non-syndromic forms of inherited optic neuropathies [8], without HL. Finally, a dominant variant has been identified in the case of an isolated nuclear congenital cataract [9]. Together, these published data indicate that *WFS1* mutations appear to underlie a spectrum of various dominant and recessive disorders, featuring a common multimodal neurosensory involvement.

Although the role of WFS1 in the occurrence of diabetes and OA is well documented, little is known about its implication in deafness. The only *post-mortem* analysis of a human inner ear demonstrated that WFS1’s deficiency led to a degeneration of the organ of Corti, a mild loss of the spiral ganglion neurons and a focal degeneration of the stria vascularis concomitantly to a symmetric high tone sensorineural HL [10]. In the murine inner ear, *Wfs1* is expressed in both auditory and vestibular systems, in a variety of cell types. In the cochlea, the protein is identified in most of the epithelial and neurosensory cells, the spiral ganglion neurons and the marginal cells of the stria vascularis [11]. In the vestibule, the protein is mainly detected in hair cells [11]. In non-human primate, WFS1 is expressed in the same cell types with the addition of the basal cells of the stria vascularis [12].

Despite recent studies unraveling Wolframin implication in the endoplasmic reticulum (ER) stress response and Ca^2+^ homeostasis [13–16], its role in the inner ear remains unclear. Early study from Cryns et al. [11] suggested a putative role of Wolframin in the ion homeostasis [11]. Strengthening this hypothesis, the Na^+^/K^+^ATPase β1 subunit was indeed the first identified molecular partner of WFS1, using a yeast-2-hybrid screen and complementary approaches [17]. The β1 subunit is a component of the sodium-potassium pump, which is composed of an assembly of one α subunit (four isoforms), one β subunit (three isoforms), and a FXDY protein (seven proteins) [18, 19]. The pump is involved in different functions in the inner ear including the ionic flux in the stria vascularis. Molecular studies showed that the β1 subunit is present at the basolateral membrane of the marginal cells of the stria vascularis in human [20, 21] and murine cochleae [22, 23].

So far, among Wolfram syndrome established animal models, hearing impairment has only been reported in the rat [24, 25], but not in zebrafish [26, 27] nor the mouse [28, 29]. *Wfs1*^*KO*^ rats develop progressive, low-frequency, neurosensory HL. In human, contrary to WS, all Wolfram-like syndrome affected individuals develop deafness. Therefore, to decipher WFS1’s role(s) in the auditory pathway, we generated a knock-in mouse model reproducing the human variant c.2590G > A, p.(E864K). The glutamate amino-acid is highly conserved across species and this previously identified disease-causing variant induces, in human, a low-frequency sensorineural HL associated with OA and glucose intolerance [4, 5] or non-syndromic low-frequency HL [30, 31]. In a previous study, we characterized the central deficits of the *Wfs1*^*ΔExon8*^ mice [16], for which no hearing exploration had been documented so far. Thus, we studied, concomitantly, the auditory function of both mutant lines, *Wfs1*^*ΔExon8*^ and *Wfs1*^*E864K*^.

Here, using physiological and morphological approaches, we report that *Wfs1*^*ΔExon8*^ homozygous mice exhibited a late onset and moderate HL while *Wfs1*^*E864K*^ homozygous mice developed a devastating vestibular syndrome and profound deafness, associated with an extremely severe degeneration of hair cells and vacuolization of the intermediate cells of the stria vascularis. The p.E864K mutation did not impact the protein interaction between WFS1 and its known interactor: the Na^+^/K^+^ATPase β1 subunit. However, the mutant protein impaired the localization of the ATPase subunit to the cell surface. Collectively our results suggest that WFS1 plays a key role in the auditory pathway, more particularly in the ionic homeostasis of the stria vascularis through its interaction with the Na^+^/K^+^ATPase β1 subunit.

## Materials and methods

### Mouse lines and ethical statement

Animal care and experimentation were authorized by the National Ethic Committee (Paris) (APAFIS #6341-2016080510211993) and carried out in strict adherence to the European Union Directive 2010/63 and ARRIVE guidelines [32]. Mice were housed in cages with *ad libitum* water and food, except during experiments. The rooms were temperature-controlled, lit on a 12:12 h light/dark cycle, lights on at 7:00 h. All functional experiments were performed during the light cycle.

*Wfs1*^*ΔExon8*^ founder mice were generated as described in [29] and kindly provided by Dr Koks (University of Tartu, Estonia). The line was maintained on a 129S6/SvEvTac × C57BL/6J - hybrid genetic background. All functional assessments were performed with both males and females and no sex-related differences were measured.

The *Wfs1*^*E864K*^ mutant mouse line was generated at the Institut Clinique de la Souris (Strasbourg, France, http://www.ics-mci.fr/en/). In brief, a targeting vector carrying the G > A transversion at position c.2590 (NM_011716.2), homologous sequences surrounding this genomic region and a floxed neomycin resistance cassette, allowing for a G418 driven cell selection, was electroporated in C57BL/6N embryonic stem (ES) cells. Once neomycin positive ES cells were isolated, long-range PCR (polymerase chain reaction) and southern blot using an internal neomycin probe and an external 5′ probe were carried out to analyze the positive clones. Among these, a unique clone was selected, karyotyped and micro-injected into BALB/c blastocysts. The chimeric males, resulting from the microinjections, were then bred with wild-type C57BL/6N females. The germline transmission was achieved in the first litter. The Neo cassette, flanked with loxP sites, was removed by crossing the first generation with a C57BL/6N CAG-Cre line. Mice were back-crossed on a C57BL/6N genetic background then intercrossed to generate wild-type, heterozygous and homozygous mutants as well as getting rid of the Cre allele. All functional assessments were performed with both males and females and no sex-related differences were measured.

Genotyping and sequencing were performed with the following primers: AAATGTGCCAGTTGGGTGACTG (forward) and GTGGAATTACCACACGTGACTG (reverse) (WT amplicon, 265 base pairs (bp); mutant amplicon, 300 bp). Genotyping was performed using routine PCR and following protocol: 95 °C for 3 min, 35 cycles (95 °C for 30 s, 63 °C for 30 s, and 72 °C for 30 s), 72 °C for 7 min. The different bands were separated on a 2% agarose gel.

For all the following studies involving mice, recordings, measurements, and analysis were performed blindly.

### Functional hearing assessments

All the following evaluations were performed under anesthesia (2% xylazine (3 mg/ml) and ketamine (40 mg/kg) intraperitoneal injection), in both male and female mice. All the evaluations were carried out in a sound-proof, Faraday-shielded, anechoic cage. The animal rectal temperature was monitored with a thermistor probe and maintained at 38.5 °C ± 1 thanks to a heated blanket placed under the anesthetized animal.

Distortion products of otoacoustic emissions (DPOAEs) and auditory brainstem responses (ABRs) were recorded at post-natal (P) day 21, P23, P25, P27, P29, and P31 of *Wfs1*^*WT*^, *Wfs1*^*E864K/+*^, and *Wfs1*^*E864K*^ mice, 4-month-old, and 10-month-old *Wfs1*^*ΔExon8*^ mice and wild-type littermates, as previously described [33]. Endocochlear potential (EPs) were measured at P31, in *Wfs1*^*WT*^, *Wfs1*^*E864K/+*^, and *Wfs1*^*E864K*^ mice.

#### DPOAEs

DPOAEs were recorded with an ER-10C S/N 2528 probe (Etymotic Research Inc., Elk Grove Village, IL, USA), in the external auditory canal of the mice. Two primary tones of frequency f_1_ and f_2_ with a constant frequency ratio f_2_/f_1_ = 1.2 were generated, and the distortion product, 2f_1_-f_2_, processed by a Cubdis system HID 40133DP (Mimosa Acoustics Inc., Champaign, IL, USA). Before each recording, the probe was self-calibrated for the two primary tones. f_1_ and f_2_ were presented simultaneously, varying f_2_ from 20 to 2 KHz by quarter octave steps. 2f_1_-f_2_, the distortion product, and the neighboring noise amplitude levels were measured and expressed as a function of f_2_, for each tested frequency.

#### ABRs

Sound stimuli, consisting of 10 ms tone-bursts with a 1 ms rise- and fall time, and delivered at a rate of 10/s, were generated by a NI PXI-4461 signal generator (National Instruments). Sound was produced by a JBL 075 loudspeaker (James B. Lansing Sound), which was at 10 cm from the tested ear in a calibrated, free-field condition. Cochlear responses were amplified (20,000) via a Grass P511 differential amplifier, and averaged 1000 times (Dell Dimensions). For each tested frequency (2, 4, 6.3, 8, 10, 12.5, 16, 20, 24, and 32 KHz), intensity-amplitude functions of the ABRs were obtained by varying, in 5 dB incremental steps, the level of the tone bursts from 0 to 100 dB SPL. The ABR thresholds were defined as the minimum sound intensity necessary to elicit well-defined and reproducible wave II.

#### Endocochlear potential (EP)

The bone of the scala media basal turn was gently shaved off, creating a small fenestra. A glass microelectrode (tip diameter 0.1–0.5 μm), filled with a 0.15 M KCl solution and connected to a direct current amplifier (WPI, model 773 A; Sarasota, FL, USA), was placed visually. The chosen position and angle were allowing the probe to pass through the fenestra and record the EP. The reference used during the recording was an Ag/AgCl reference electrode, placed in the neck musculature of the mouse.

### Balance assessments

#### Rotarod

A rotarod apparatus (Bioseb, Chaville, France) was used to test the balance and motor coordination of *Wfs1*^*E864K*^ mice. Ten to fifteen *Wfs1*^*WT*^ and *Wfs1*^*E864K*^ mice were included in this assessment. Familiarization of the mice with the rotating rod was immediately followed by a training session on day 1 (P24). The mice were trained to stay on the rod, with a progressively increased speed (from 4 rpm up to 10 rpm). Following each fall, the mice were replaced on the rotating rod (total of 4 trials on day 1). On day 2 (P25) and 3 (P26), the mice were tested with a fixed speed of 10 rpm. During these trials, the latency to fall was measured up to 180 s. Each animal underwent 4 trials per day. For each training and test day, the data were averaged per mouse and subsequently per group (*Wfs1*^*WT*^ and *Wfs1*^*E864K*^ homozygous mice).

#### Behavioral experiments

We established a vestibular rating score for each mouse, estimated as previously described [34, 35]. *Wfs1*^*WT*^ and *Wfs1*^*E864K*^ homozygous mice were scored, individually, from 0 to 4, at P21, P24, P27, and P30. A score of 0 means that behavior is normal; a score of 1 means an abnormal behavior without any specific vestibular deficit; a score of 2 describes an identified but mild vestibular deficit; a score of 3 represents an identified and manifest deficit; and a score of 4 is given to a maximal vestibular deficit.

Six different tests, described below, were sequentially scored and averaged to rate the vestibular deficit of each individual mouse. (1) Head bobbing corresponds to intermittent, abnormal, backward extension of the neck. (2) Circling stereotyped movement was scored, ranging from none to compulsive circles around the animal’s hips. (3) The tail-hanging reflex, which induces a forelimb extension to reach the ground in WT animals, results, when the vestibular deficit is maximal, in the ventral bent of the body and grip of the tail. (4) The contact-inhibition reflex leads the animal to hold on to a metal grid in a supine position to return when its back touches the ground. Without a body orientation referential due to vestibular deficit, this reflex is abolished, and the animal keeps on gripping the grid in a supine position. (5) The air-righting reflex allows the animals, when they fall from a prone position, to roll over and land on their feet; this normal reversal is impaired when animal exhibits a vestibular alteration. When dropped from a height of 40 cm onto a foam cushion, animals with a maximal deficit land on their back. (6) Swimming ability was scored from normal swimming behavior to drowning due to loss of all proprioceptive clues.

The data were averaged per mouse and subsequently per group (*Wfs1*^*WT*^ and *Wfs1*^*E864K*^ homozygous mice).

### Morphological assessments

The ultrastructural characteristics of the cochlear neurosensory epithelium in 10-month-old *Wfs1*^*ΔExon8*^ mice and wild-type littermates and P23, P27, and P31 *Wfs1*^*WT*^ and *Wfs1*^*E864K*^ homozygous mice were analyzed using confocal microscopy (LSM880, Fast Airyscan, Zeiss) after immunohistochemistry. Additional analyses were performed by scanning electron microscopy (SEM) using a Hitachi S4000 microscope and transmission electron microscopy (TEM) with a JEOL 1400 microscope.

The ultrastructural characteristics of the vestibular neurosensory epithelium in P23, P27, P31, and P66 *Wfs1*^*WT*^ and *Wfs1*^*E864K*^ homozygous mice were analyzed using SEM.

#### Cochlear sensory hair cells

Sensory hair cell and stereocilia abnormalities of *Wfs1*^*ΔExon8*^, *Wfs1*^*WT*^, and *Wfs1*^*E864K*^ homozygous mice were evaluated using SEM. Previously reported techniques were used to process and evaluate the cochlea sensory hair cells [36]. Inner and outer hair cells from the apical (0.5–1 mm from the apex tip, corresponding to the 6–8 KHz region), middle (1.9–3.3 mm from the apex tip, 12–24 KHz region), and basal (4.1–5.0 mm from the apex tip, 32–50 KHz region) segments of the cochlea were imaged.

#### Spiral ganglion neurons and Stria vascularis

The cochleae from *Wfs1*^*WT*^ and *Wfs1*^*E864K*^ homozygous P31 mice were harvested, perfused through the round and oval windows, and fixed for approximately 30 min at room temperature (RT) with 10% formalin diluted in phosphate-buffered saline (PBS). The cochleae were decalcified in 0.2 M EDTA in PBS for 2 days before further processing. Cochleae were then placed in 10% sucrose in PBS, 2:1 10%:30% sucrose, 1:1 10%:30% sucrose, 1:2 10%:30% sucrose, for 30 min at room temperature each time, 30% sucrose in PBS overnight at 4 °C and Tissue-Tek OCT (Miles, Diagnostics Division, Elkhart, IN, USA) for 1 h at room temperature. Cryosections of OCT-mounted cochleae were cut at 14 μm.

After 1 h blocking with 10% Horse serum (HS) in 0.1% Triton X-100/PBS at room temperature, cochlear cryosections were immunostained at 4 °C, overnight, with anti-Peripherin (1:200, Rabbit, Millipore, Cat#AB1530, RRID:AB_90725) and anti-Tubulin beta 3 (TUJ1) (1:400, Mouse, BioLegend Cat# 801201, RRID:AB_2313773), to label the type II and types I and II spiral ganglion neurons, respectively. For stria vascularis analysis, cochlear cryosections were immunostained, overnight at 4 °C, with anti-Claudin 11 (1:200, Rabbit, Thermo Fisher Scientific Cat# 36-4500, RRID:AB_2533259) anti-Kir4.1 (1:300, Rabbit, Alomone Labs Cat# APC-035, RRID:AB_2040120), anti-KCNQ1 (1:200, Mouse, Santa Cruz Biotechnology Cat# sc-365186, RRID:AB_10707688) and anti-NKCC1 (1:200, Rabbit, Millipore Cat# AB3560P, RRID:AB_91514) to label the different cell layers of the stria vascularis. The sections were then washed in 0.1% Triton X-100/PBS three times, incubated for 1 h at room temperature with secondary antibody solution (Alexa-488 conjugated goat anti-mouse IgG (1:500); Alexa-546 conjugated goat anti-rabbit IgG (1:500); Invitrogen) with 3% HS in 0.1% Triton X-100/PBS. Nuclei were counterstained using 4’,6-diamidino-2-phenylendole (DAPI; 1:5000, Sigma-Aldrich). Cryosections were rinsed three times with 0.1% Triton X-100/PBS, three more times with PBS and mounted on glass slides with ProlongGold Antifade mounting medium (Invitrogen).

All images were acquired using a Zeiss LSM880 Fast-airy-scan scanning confocal microscope. The cross-sectional area of Rosenthal’s canal as well as the number of SGN were measured, using the NIH ImageJ software. The number of neurons was then divided by the cross-sectional area to determine the SGN density (*n* = 4 to 5 sections per cochlea, 3 to 4 cochleae per genotype).

#### Ultrastructural analysis: transmission electron microscopy (TEM)

Morphological changes were investigated using TEM analyses. *Wfs1*^*WT*^ and *Wfs1*^*E864K*^ homozygous mice were perfused, intracardially, with a 4% formaldehyde solution in Sorensen’s buffer (19% NaH_2_PO_4_; 81% Na_2_HPO_4_; pH 7.4). For each mouse, both inner ears were extracted and incubated in 2.5% glutaraldehyde in Sorensen buffer for 2 h at 4 °C, washed in Sorensen buffer and postfixed in 2% osmium tetroxide for 1 h at room temperature, in the dark. After three washes in Sorensen buffer, the inner ears were dehydrated in a graded series of ethanol solutions (30 to 100%) then embedded in Epon. Ultrathin sections of 70 nm, using a Leica-Reichert Ultracut E, were collected from different levels of each block. These sections were examined in JEOL 1400 TEM at 80 kV, after being stained with uranyl acetate and lead citrate. To document and evaluate the vacuolization process in the different cell types of the cochleae, we photographed different sub-structures (Spiral Ganglion Neurons, Stria Vascularis, hair cells) for each sample with a 11 MegaPixel bottom-mounted TEM camera system (Quemesa, Olympus). iTEM software (Olympus SIS) was used to analyze the images.

### Molecular assessment

#### Constructs

Wild type (WT) human *WFS1* cDNA (pCMV-Myc-WFS1) and human *ATP1B1* (pCMV-HA-ATP1B1) were kindly gifted by Dr Barrett [17]. The G > A transversion at cDNA position 2590 (NM_001145853.1) into WT *WFS1* sequence was engineered by GeneScript and termed WFS1^E864K^.

#### Cell culture

Human embryonic kidney 293 cells (HEK293T) and Madin-Darby Canine Kidney cells (MDCK) were purchased from the ATCC (atcc.org). They were not recently authenticated nor tested for mycoplasma contamination. They were cultured in a high glucose and Glutamax supplemented Dulbecco’s modified Eagle’s medium (#31966-021, Sigma-Aldrich, Saint Louis, Missouri, USA) complemented with 10% fetal bovine serum (FBS) (Sigma-Aldrich) and 20 mM penicillin/streptomycin (50 U/ml, Sigma-Aldrich) and maintained at 37 °C in 5% CO_2_.

#### Cell transfection

HEK293T cells (10 cm plates) and MDCK cells (6 well plates) were transfected at 80–90% confluency with 10 μg/plate per construct (HEK) or 2 μg/well per construct (MDCK), after mixing with polyethyleneimine (PEI-MAX; Polysciences, Inc., Warrington, PA, USA) at a 1:3 ratio and 1:5 ratio of DNA:PEI in DMEM medium (Sigma-Aldrich), respectively. The DNA:PEI mixture was added to cells, maintained in DMEM medium that contained 2% FBS and 20 mM penicillin/streptomycin and grown for 2 days for HEK cells. MDCK cells were cultured for an additional 72 h prior to immunostaining.

#### Co-immunoprecipitation (co-IP)

HEK293T cells were co-transfected with HA-tagged ATP1B1 and Myc-tagged WFS1 (WT or E864K) constructs, using PEI-MAX reagent. Cells were harvested 48 h after transfection and the pellets were homogenized and sonicated in 600 μl of lysis buffer (50 mM Tris HCl pH 7.4, 150 mM NaCl, 1 mM EDTA, 3 mM EGTA, 1 mM DTT, 1% NP-40, 0.1% SDS, 0.1% DOC) containing protease inhibitors (Complete mini, Roche) and phosphatase inhibitors (PhosSTOP, Sigma). 50 µl of protein G sepharose beads (protein G sepharose 4 Fast Flow, Cytiva) were prepared according to the manufacturer’s instructions and added to each lysate, incubated overnight on a wheel, at 4 °C. Immunoprecipitation was performed with 10 μg of a monoclonal mouse anti-HA (Biolegend, RRID: AB_2148451), at 4 °C for 2 h, on a wheel. The samples were centrifuged for 5 min at 500 rpm and washed 3 times with 1 ml of ice-cold lysis buffer. The pellets obtained after the last wash were resuspended in 50 µl of Laemmli sample buffer and heated for 5 min at 95 °C. For western blotting, the supernatants and the cell lysates were loaded on SDS-PAGE gels. HA-tagged ATP1B1, Myc-tagged WT WFS1 and WFS1 E864K simple transfections were used as controls.

#### Biotinylation assay

Cell surface biotinylation assays were performed as reported previously [37]. Briefly, HEK293T cells were co-transfected with HA-tagged ATP1B1 and Myc-tagged WFS1 (WT or E864K) constructs, using PEI-MAX reagent. Ten cm HEK cell plates were washed, 48 h after transfection, three times with ice-cold 100 µM CaCl_2_, 1 mM MgCl_2_ in PBS. The EZ-Link Sulfo-NHS-SS-Biotin (# 21331, ThermoFisher) (1.5 mg/ml in 10 mM triethanolamine pH 9.0, containing 2 mM CaCl_2_ and 150 mM NaCl) was used to biotinylate the surface proteins for 25 min, on ice, two times. Cells were then washed twice with ice-cold 100 µM CaCl_2_, 1 mM MgCl_2_, 100 mM glycine in PBS and quenched with the same buffer for 20 min on ice. After two additional washes in ice-cold 100 µM CaCl_2_, 1 mM MgCl_2_ in PBS, the cells were lysed with a specific lysis buffer (1% Triton X-100, 150 mM NaCl, 5 mM EDTA, 50 mM Tris-HCl, pH 7.5), on ice, for 1 h. Cell lysates were centrifuged at 14,000 × *g* for 10 min at 4 °C. Biotinylated proteins were precipitated with a 50% slurry of Streptavidin Agarose beads (# 20353, ThermoFisher) overnight, at 4 °C. The beads were then washed twice with high-salt (0.1% Triton X-100, 500 mM NaCl, 5 mM EDTA, 50 mM Tris HCl, pH 7.5) then no-salt (50 mM Tris HCl, pH 7.5) washing buffers. Beads were incubated at 55 °C for 30 min in 100 µl 2x Laemmli buffer. The supernatants and cell lysates were loaded on SDS-PAGE gels for western blotting. HA-tagged ATP1B1, Myc-tagged WT WFS1, and WFS1 E864K simple transfections were used as controls.

#### Western blot analysis

For co-IP and biotin assays, proteins of the supernatants and cell lysates were separated, based on their molecular weights, on SDS-polyacrylamide gels and electroblotted onto nitrocellulose membranes (Bio-Rad). Membranes were saturated with 5% non-fat milk in 0.1% Tween/PBS for 1 h at room temperature. Incubation of the membranes with primary antibodies was carried out overnight at 4 °C with monoclonal mouse anti-HA (1:1000, Biolegend, RRID: AB_2148451), and anti-Myc (1:1000, Biolegend, RRID: AB_2565336). The following day, after three washes in 0.1% Tween/PBS, the membranes were incubated with anti-mouse IgG horseradish peroxidase linked antibody (1:2000, Abcam) for 1 h at room temperature in 5% non-fat milk dissolved in 0.1% Tween/PBS. The proteins were detected with enhanced chemiluminescence (ECL + Western Blotting Detection Reagents, Amersham Biosciences, UK) using the BioRad ChemiDoc2 Touch Imaging System.

#### Immunofluorescence

MDCK cells, 72 h post transfection, were rinsed with PBS, fixed in 4% paraformaldehyde at room temperature for 20 min and blocked with 10% HS in 0.1% Triton X-100/PBS. The cells were then incubated with primary antibody (1,1000 anti-Myc, Biolegend; 1,1000 anti-HA, Biolegend) with 3% HS in 0.1% Triton X-100/PBS, overnight at 4 °C. The following day, the cells were washed in 0.1% Triton X-100/PBS and incubated, for 1 h at room temperature, with the Alexa-546 and Alexa-488 conjugated secondary antibodies (Life Technologies) with 3% HS in 0.1% Triton X-100/PBS. Filamentous actin was stained with Alexa-647 phalloidin (ThermoFisher) in 0.1% Triton X-100/PBS for 1 h. The cells were then washed and the coverslips were mounted on glass slides, using FluoroGel mounting medium (Electron Microscopy Sciences, Hatfield, PA, USA).

All images were acquired using a Zeiss LSM880 Fast Airyscan scanning confocal microscope equipped with 63x objective, and the analyses were performed using ImageJ software.

### Statistical analyses

Data are expressed as mean ± standard error to the mean (SEM). No statistical methods were used to predetermine the sample size. No outlier was discarded during statistical analysis.

All mice were age-matched and then randomized into different groups. Recordings, measurements, and analyses were performed blindly.

**Fig. 1:**
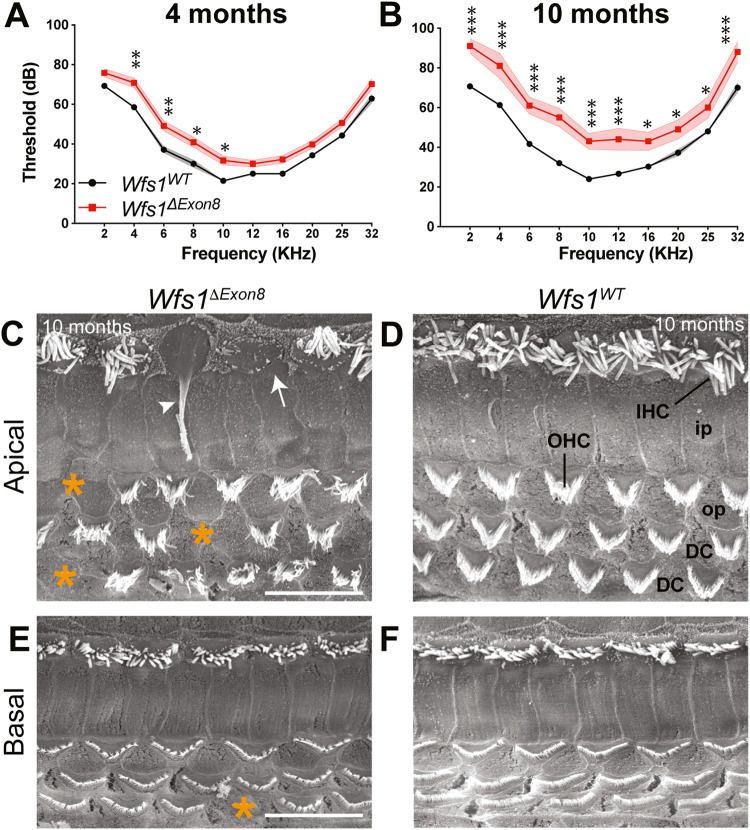
*Wfs1*^*ΔExon8*^ mice have late onset and progressive hearing loss. **A**, **B** Averaged auditory brainstem responses (ABR) thresholds of homozygous *Wfs1*^*ΔExon8*^ mice (red) and their control littermates (*Wfs1*^*WT*^, black) at 4 (**A**) and 10 (**B**) months in response to 2, 4, 6, 8, 10, 12, 16, 20, 25, and 32 KHz tone-bursts, with sound pressure level (SPL) of 0 to 100 dB. The *Wfs1*^*ΔExon8*^ mice showed statistically significant elevated thresholds for all the lowest tested frequencies (2 to 10 KHz) compared to their control littermates at 4 months. By 10 months, the deficits worsened and the mutant mice showed statistically significant elevated thresholds for all tested frequencies (two-way ANOVA test was followed by *Bonferroni’s* test, *p < 0.05; **p < 0.01; ***p < 0.001, mean ± SEM; for 4 months mice, *n* = 13 *Wfs1*^*ΔExon8*^, *n* = 7 *Wfs1*^*WT*^; for 10 months mice, *n* = 5 *Wfs1*^*ΔExon8*^, *n* = 11 *Wfs1*^*WT*^*)*. **C**–**F** Scanning electron micrographs of the organ of Corti from the apical (**C**) and basal (**E**) turns of *Wfs1*^*ΔExon8*^ mice and their control littermates (*Wfs1*^*WT*^) at 10 months (**D**, **F**). Some outer hair cells are missing from both regions of the neurosensory epithelium, as shown with the asterisks (*). Inner hair cells are still present, however, in the apical turn, some stereocilia bundles show fused and elongated stereocilia (arrowhead) or degenerated stereocilia (white arrow). IHC: inner hair cell, OHC: outer hair cell, ip: inner pillar cell, op: outer pillar cell, DC: Deiters’ cell. Scale bar: 15 μm for all images.

**Fig. 2:**
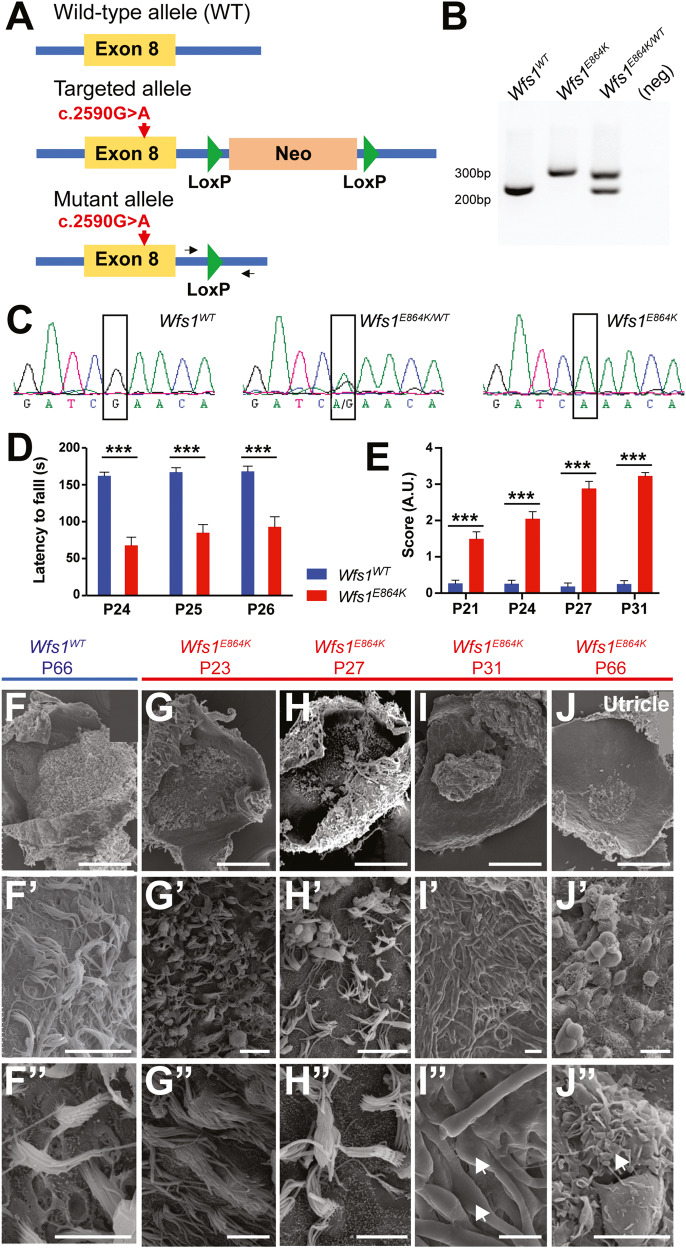
*Wfs1*^*E864K*^, a new mouse model for Wolfram syndrome, develops severe vestibular deficits. **A** Schematic of the structure of the wild type, targeted and mutant alleles. The red arrows point out the localization of the p.E864 residue. PCR primers are represented as black arrows. **B** Representative PCR genotyping results of *Wfs1*^*WT*^*, Wfs1*^*E864K*^, and *Wfs1*^*E864K/+*^ mice. H_2_O was used as negative control (neg). **C** Sanger sequencing chromatograms confirming the G > A transversion at position c.2590 in *Wfs1*^*E864K/+*^ and *Wfs1*^*E864K*^ mice. **D** Comparative analysis of the time spent on fixed speed rotating rod (10 rpm) before falling (latency to fall in seconds) for P24, P25, and P26 *Wfs1*^*WT*^ (blue; *n* = 14) and *Wfs1*^*E864K*^ (red; *n* = 15) mice. Mutant mice showed impaired performance compared to *Wfs1*^*WT*^ mice. Each mouse was subjected to 4 trials of 180 s duration per day. Data are mean ± SEM (two-way ANOVA followed by Tukey’s test, ***p < 0.001). **E** Evaluation of vestibular deficits in P21, P24, P27, and P31 *Wfs1*^*WT*^ (blue; *n* = 15) and *Wfs1*^*E864K*^ (red; *n* = 13) mice. Six different tests were evaluated (scored from 0 to 4) and averaged to obtain a vestibular deficit index (in arbitrary units (AU)). *Wfs1*^*E864K*^ mice exhibited strong, progressing, vestibular deficits, statistically different from vestibular behavior of their WT littermates. Data are mean ± SEM (two-way ANOVA followed by Tukey’s test, ***p < 0.001). **F**–**J** Representative scanning electron micrographs of the sensory epithelium of the utricle of P23, P27, P31, and P66 *Wfs1*^*E864K*^ mice and P66 *Wfs1*^*WT*^ mice. Higher magnification (**F**’–**J**’, **F**”–**J**”) showed that the sensory epithelium degenerated rapidly in the mutant mice with abnormal stereocilia. White arrows point out fused and elongated stereocilia (**I**”, **J**”). Scale bars: 150, 10, and 5 μm from top to bottom.

**Fig. 3:**
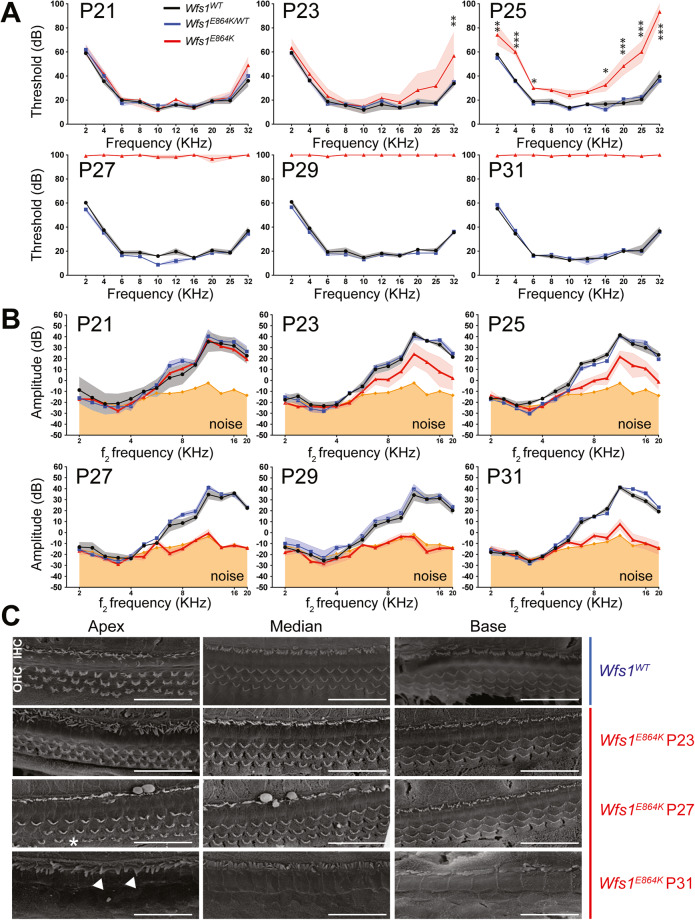
*Wfs1*^*E864K*^ mice are profoundly deaf by P27 and their hearing loss is associated with hair cell degeneration. **A** Averaged auditory brainstem responses thresholds of homozygous *Wfs1*^*E864K*^ mice (red) and their control littermates (*Wfs1*^*E864K/+*^ (blue) and *Wfs1*^*WT*^ (black)) at P21, P23, P25, P27, P29, and P31 in response to 2, 4, 6, 8, 10, 12, 16, 20, 25, and 32 KHz tone-bursts, with sound pressure level (SPL) of 0 to 100 dB. The *Wfs1*^*E864K*^ mice showed statistically significant (*p* < 0.001) elevated thresholds for all the tested frequencies compared to their control littermates by P27 (two-way ANOVA followed by Tukey’s test, *p < 0.05; **p < 0.01; ***p < 0.001, mean ± SEM; *n* = 9 *Wfs1*^*E864K*^, *n* = 9 *Wfs1*^*E864K/+*^ and *n* = 7 *Wfs1*^*WT*^). **B** Distortion product otoacoustic emission (DPOAE) amplitudes of *Wfs1*^*E864K*^ (red), *Wfs1*^*E864K/+*^ (blue) and *Wfs1*^*WT*^ (black) at P21, P23, P25, P27, P29, and P31, represented as a function of f_2_ stimulus frequencies. By P27, *Wfs1*^*E864K*^ mice showed attenuated responses to no responses, with values close to the mean noise floor levels (orange) (mean ± SEM; *n* = 9 *Wfs1*^*E864K*^, *n* = 9 *Wfs1*^*E864K/+*^ and *n* = 7 *Wfs1*^*WT*^). **C** Scanning electron micrographs of the organ of Corti from the three regions of *Wfs1*^*E864K*^ mice and their control littermates (*Wfs1*^*WT*^) at P23, P27, and P31. While no degeneration is seen at P23 and P27, by P31 all the outer hair cells (OHC) are absent. Inner hair cells (IHC) are still present, however, some stereocilia bundles show morphology deficit with fused stereocilia, as pointed out by the white arrowheads. Scale bar: 30 μm for all images.

Prism v.7.0 software (GraphPad, San Diego, CA) was used for all statistical analyses. The software systematically performs a Bartlett’s test which is enabling us to select the correct statistical test, parametric tests in this study. Unpaired Student’s t test or two-way ANOVA were performed to determine statistical significance between groups. The levels of statistical significance were: *p < 0.05, **p < 0.01, and ***p < 0.001. Two-way ANOVA statistical values for Fig. 1 (Table S1), Fig. 2 (Table S2), and Fig. 3 (Table S3) are available in the supplemental information.

## Results

### *Wfs1*^*ΔExon8*^ mouse model developed a late onset hearing deficit

To assess the effect of *Wfs1* deletion on hearing, we used a previously characterized mouse model, the *Wfs1*^*ΔExon8*^ line, and recorded the auditory brainstem responses (ABRs) in *Wfs1*^*ΔExon8*^ homozygous mice and their wild-type littermates. ABR thresholds measured during our functional exploration in *Wfs1*^*ΔExon8*^ mice were comparable to those of the *Wfs1*^*WT*^ (WT) mice at 4 months, to the exception of a 10 dB difference in the lowest frequencies, up to 10 KHz included (Fig. 1A). These differences worsened with age and led to a mild to moderate hearing deficit by 10 months of age. At all tested frequencies, significantly higher (10 to 20 dB) ABR thresholds were measured in *Wfs1*^*ΔExon8*^ mice compared to control mice (Fig. 1B). This impairment is associated with moderate outer hair cell loss as well as morphological abnormalities of inner hair cell stereocilia throughout the cochlea (Fig. 1C–F).

### Generation of the *Wfs1*^*E864K*^ mouse and characterization of vestibular deficits

To better understand WFS1 role(s) in the inner ear, we generated a *Wfs1*^*E864K*^ mouse model thus presenting the identified human WFS1^E864K^ mutation [4, 5, 30, 31], following a classical protocol of electroporation of a vector containing the genomic DNA of mouse exon 8 with the transition mutation G to A at position c.2590 (NM_011716.2), in ES cells (Fig. 2A). Mice carrying the mutant allele were crossed with CAG-Cre mice to remove the neomycine cassette and their offspring were intercrossed to generate control, heterozygous and homozygous mice (Fig. 2B). The presence of the variant was validated by Sanger sequencing (Fig. 2C).

Surprisingly, the mutant mice developed overt vestibular deficits (Fig. 2D, E), starting at post-natal day 21 (P21). Using fixed speed rotarod analysis, the latency to fall of the *Wfs1*^*E864K*^ mutant mice was significantly shortened when compared with their WT littermates (Fig. 2D). To assess the severity and the evolution of the vestibular alterations, several behavioral tests were performed, including circling, head bobbing, tail-hang reflex, contact inhibition reflex, swimming, and air-righting reflex tests at P21-P31 (Figure S1), out of which a global score was calculated (Fig. 2E). These analyses confirmed a rapid and severe progression of the mutants balance dysfunction. To determine the cause of this deficit, we investigated the morphology of the vestibular epithelium using scanning electron microscopy (Fig. 2F–J”). At P23, the epithelium appeared normal and well organized (Fig. 2G–G”) but the stereocilia bundles gradually fused and degenerated (Fig. 2H–H”) leading to a complete loss of the vestibular epithelium by P31 in the *Wfs1*^*E864K*^ mutant mice (Fig. 2I–J”). The increase of the balance deficits of the mutant mice was timely correlated to the degradation of the vestibular hair cells, suggesting that the hair cell loss is partially responsible of the vestibular syndrome. However, the difference in onset between the vestibular dysfunction and the hair cell loss might suggest that hair cell loss is not the sole cause of the deficit.

### *Wfs1*^*E864K*^ mice showed severe hearing deficits with a massive and abrupt hair cell degeneration

Concomitantly to vestibular characterization, to examine the effect of the *Wfs1*^*E864K*^ variant on the cochlear function, we measured sound-evoked ABR in *Wfs1*^*E864K*^ mutant mice and their control littermates (*Wfs1*^*E864K/+*^ and *Wfs1*^*WT*^ mice). At P21, normal ABR thresholds and waveforms were measured in all *Wfs1*^*WT*^, *Wfs1*^*E864K/+*^, and *Wfs1*^*E864K*^ mice. However, *Wfs1*^*E864K*^ mutant mice exhibited a rapid flattening of their ABR waveforms and an elevation of their ABR thresholds (Fig. 3A). While the hearing deficit started in the high frequencies (P23), it rapidly gained all tested frequencies to lead to a complete loss of hearing at all tested frequencies by P27-P29.

To better characterize the hearing deficit, we also recorded the distortion product otoacoustic emission (DPOAE), a by-product of cochlear amplification dependent on outer hair cells (OHC) integrity. As observed for ABR measurements, *Wfs1*^*E864K/+*^ and *Wfs1*^*WT*^ mice presented with normal and similar DPOAEs while those of the mutant mice were rapidly not distinguishable from the noise floor, at P27-P29 (Fig. 3B). Note that the time course and magnitude of the increase in ABR thresholds is very similar to that of the decrease in DPOAE amplitude during the first post-natal month in mutant mice. Thus, these results suggest that hearing loss in *Wfs1*^*E864K*^ mutant mice might be in part explained by OHC alteration.

Based on these physiological data, we closely examined the neurosensory epithelium of the organ of Corti at the same stages (Fig. 3C). At P23, the row of inner hair cells (IHCs) appeared well formed and normal, and no morphological differences were seen in the three rows of OHCs between mutant and control mice. At P27, protrusions were observed at the base of the stereociliary bundles of the IHCs and OHCs at the apical and medial turns of the cochleae of the *Wfs1*^*E864K*^ mice. In addition, some OHCs were missing from the neurosensory epithelium in the apical part of the mutant cochleae. By P31, IHCs presented with fused stereocilia bundles and all three rows of OHCs were missing.

### *Wfs1*^*E864K*^ mice did not present overt spiral ganglion neuron alteration at early stages

**Fig. 4:**
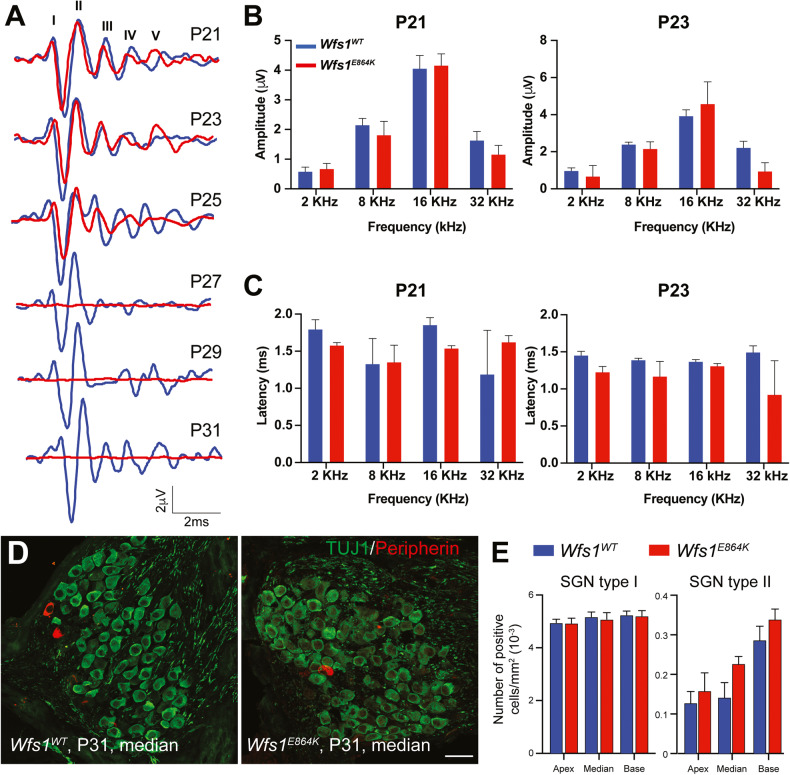
Hearing loss of *Wfs1*^*E864K*^ mouse is not primarily due to SGN alteration. **A** Average ABR waveform evoked by 16 KHz tone-bursts at 70 dB SPL in P21 to P31 *Wfs1*^*E864K*^ (red) and *Wfs1*^*WT*^ (blue) mice. Roman numbers (I–V) indicate the five vertex positive waves. Wave 1 amplitudes (**B**) and latencies (**C**) are not altered at P21 and P23, the two time points tested before any hearing deficit onset. Data are represented as the mean ± SEM (*n* = 9 *Wfs1*^*E864K*^, *n* = 9 *Wfs1*^*E864K/+*^ and *n* = 7 *Wfs1*^*WT*^). **D** Representative confocal images of Rosenthal’s canal in the middle turn of the cochlea. Spiral ganglion neurons of *Wfs1*^*WT*^ and *Wfs1*^*E864K*^ mice at P31, were immunolabeled for TUJ1 (green) and Peripherin (red). Scale bar: 150 μm. **E** Average type I (TUJ1) and type II (Peripherin) spiral ganglion neuron densities in the apical, middle, and basal parts of the cochlea are similar between mutant mice and their control littermates at P31. Data are represented as mean ± SEM (*n* = 4 *Wfs1*^*WT*^; *n* = 3 *Wfs1*^*E864K*^).

It is generally admitted that ABR wave I is caused by the spiral ganglion neurons (SGN) of the auditory nerve, which connect hair cells to the cochlear nucleus in the auditory pathway, its amplitude and latency being therefore indicators of a potential alteration of the SGN [38]. To note, nor the amplitude or the latency of ABR wave 1 were altered in *Wfs1*^*E864K*^ mice, in early ages (P21-P23), before ABR thresholds increase, suggesting that the spiral ganglion neurons are not initially impacted by the mutation (Fig. 4A–C).

We also analyzed the impact of the *Wfs1*^*E864K*^ variant on SGN survival in *Wfs1*^*E864K*^ mice cochlear cryosections. Using fluorescent immunolabeling on slices longitudinal to the modiolus, type I and type II SGN density was determined at P31 for the spiral ganglia divided into 3 segments: basal, middle and apical segments (Fig. 4D). For each segment, the number of cell bodies for type I and type II SGN were similar between control and mutant mice (Fig. 4E) suggesting that SGN alteration is not the primary cause of *Wfs1*^*E864K*^ mice hearing loss.

### *Wfs1*^*E864K*^ mice displayed impaired endocochlear potential and huge stria vascularis atrophy

**Fig. 5:**
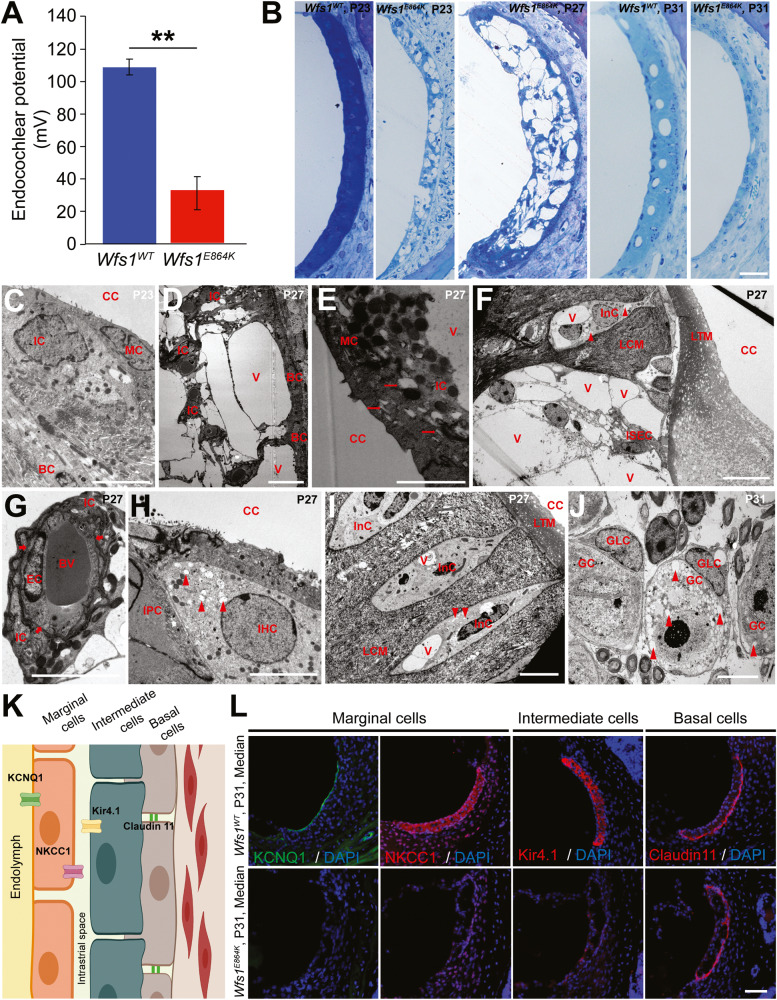
Hypertrophy and vacuolization of stria vascularis cells of *Wfs1*^*E864K*^ mice lead to a complete collapse of the SV structure by P31. **A** Measurements of the endocochlear potential magnitude showed a significant 70% reduction in the EP in P31 *Wfs1*^*E864K*^ mice (red, 32.25 ± 10.3 mV) compared with age matched *Wfs1*^*WT*^ mice (blue, 108.8 ± 4.8 mV). Data are presented as mean ± SEM (*Wfs1*^*WT*^ *n* = 6 and *Wfs1*^*E864K*^ *n* = 4; Mann–Whitney’s test, **p < 0.01). **B** Representative semi-thin sections of median coil stria vascularis from *Wfs1*^*WT*^ and *Wfs1*^*E864K*^ at different time points (P23, P27, and P31) showing the intense vacuolization of the cells leading to a complete collapse of the stria vascularis in the mutant mice as early as P31. Scale bar: 50 µm. **C**–**E** Representative transmission electron micrographs of the stria vascularis of *Wfs1*^*E864K*^ mice, at P23 and P27. At P27, the intermediate cells (IC) contain very large intracytoplasmic vacuoles (V). Such vacuolation does not occur in the marginal (MC) and basal (BC) cells. However, in the marginal cells, dilated rough ER is observed (red arrows, **E**). CC: cochlear canal. Scale bar, from left to right: 5, 10, and 2 μm. **F**–**J** Representative transmission electron micrographs of the **F** inner sulcus, **G** blood vessel in contact with endothelial and intermediate cells, **H** inner hair cell at P27, **I** interdental cells, and **J** spiral ganglion neurons at P31. To the exception of blood vessels, which are well preserved, all the other structures contain small (arrowheads) or large (V) intracytoplasmic vacuoles. The basal membrane around the blood capillary is continuous (arrows, **G**). BV: blood vessel, CC: cochlear canal, EC: endothelial cell, GC: spiral ganglion cell, GLC: spiral glial cell, IHC: inner hair cell, InC: interdental cell, IPC: inner pilar cell, ISEC: epithelial cell of the inner sulcus, LTM: limbal tectorial membrane, LCM: connective matrix of the limbus spiralis. Scale bar: 5 μm for all panels. **K** Schematic of the murine stria vascularis depicting the different cell types and the localization of the proteins used in panel (**L**) to identify these cell types. **L** Representative images of cryosections of median turn of the organ of Corti, centered on the stria vascularis of *Wfs1*^*E864K*^ and *Wfs1*^*WT*^ mice at P31. Marginal cells were immunolabeled with NKCC1 (red) and KCNQ1 (green). Intermediate and basal cells were immunolabeled with Kir4.1 (red) and Claudin 11 (red), respectively. At P31, only Claudin 11 expression could be observed in the mutant mice, suggesting that in both intermediate and marginal cell types the membrane proteins NKCC1, KCNQ1, and Kir4.1 are lost. Nuclei were marked with DAPI (blue). Scale bar: 50 μm.

The OHC degeneration was not sufficient to explain the severe hearing loss measured in the *Wfs1*^*E864K*^ mutant mice, suggesting that another structure might also be responsible. The SGN, the organ of Corti and the stria vascularis (SV) are considered to be the three major cochlear structures responsible for hearing impairment. We therefore investigated the stria vascularis function by recording endocochlear potential (EP) in *Wfs1*^*E864K*^ mutant mice and their control littermates (Fig. 5A). At P31, a statistically significant decreased of the *Wfs1*^*E864K*^ mutant mice EP was observed, when compared to WT mice EP (*p* < 0.001). Thus, the hearing loss and outer hair cell dysfunction measured in *Wfs1*^*E864K*^ mutant mice can be partially explained by this reduction in EP.

We examined the morphology of the stria vascularis at different stages (Fig. 5B–J). In a first step, we studied the morphology of the cochlea from P21 to P31 in mutant mice on semi-thin sections (Fig. 5B). Starting at P23, empty spaces, different from capillary lumen, appeared in the stria vascularis of mutant mice. They became very abundant in later stages giving the appearance of a swollen and vacuolized stripe. However, the nuclei of the various cells of the stria were still present and no sign of condensation of the chromatin was visible. At P31, all the cells collapsed to a thinner strip of cells, free of large vacuoles, where we could notice the presence of different cell layers (Fig. 5B).

To better understand the structural effects of the variant on the organization of the stria vascularis cells, we examined the same stages under transmission electron microscope (Fig. 5C–J). As early as P23 (Fig. 5C), some intermediate cells of the stria vascularis exhibited numerous small vacuoles within their cytoplasm. The vacuolation of intermediate cells increased considerably in later stages (Fig. 5D–F) and huge vacuoles occupied most of the cell volume. Such vacuolation did not occur in the marginal nor the basal cells of the stria vascularis, even though, in the marginal cells, dilated rough ER was observed (Fig. 5E). In addition, the fibrocytes of the tissue on which the stria vascularis rests were not affected by the mutation. The morphology of the blood vessels included in the stria was preserved as well as the basal membrane surrounding it (Fig. 5G). On the other hand, the organ of Corti and the spiral ganglion neurons seemed to be much less affected by the mutation. The different cells of the hearing organ (sensory and supporting cells) were clearly identifiable, and their structure seems well preserved, with the exception of small vacuoles observed in the apical part of the IHC (Fig. 5H). Only the epithelia bordering the interior or exterior of the organ of Corti, including inner and outer sulcus, interdental and Reissner’s membrane cells, were disturbed and showed the presence of many vacuoles from P27 stage (Fig. 5I and results not shown). As for the spiral ganglia, the neurons and glial cells were clearly visible. Their structure seemed to be well preserved, and very few neurons contain many small intracytoplasmic vacuoles at the most advanced stages (Fig. 5J).

Fluorescent immunolabeling with specific markers of marginal (KCNQ1, NKCC1), intermediate (KCNJ10), and basal (Claudin 11) cells of the stria vascularis (Fig. 5K, L) confirmed that, by P31, only Claudin 11 positive cells subsisted in mutant mice stria vascularis (Fig. 5K, L).

### WFS1^E864K^ protein altered Na^+^/K^+^ATPase β1 subunit targeting to the cell surface

**Fig. 6:**
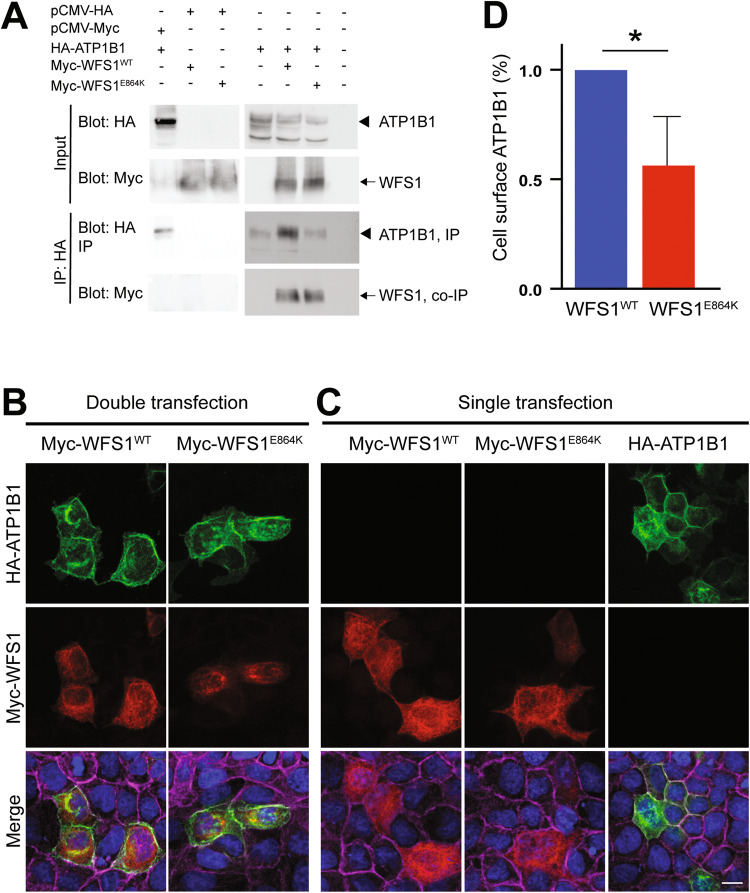
WFS1^E864K^ mutant protein interacts with the Na^+^/K^+^ATPase β1 subunit and impacts its localization to the plasma membrane. **A** Co-immunoprecipitation of WFS1 and Na^+^/K^+^ATPase β1 subunit (ATP1B1) with anti-HA antibodies. HEK293T cells were transiently transfected with Myc-tagged WFS1 and HA-tagged ATP1B1 constructs to assess the co-immunoprecipitation of WFS1 and Na^+^/K^+^ATPase β1 subunit (ATP1B1), using anti-HA antibodies. Total lysates (input) and precipitates were immunoblotted with antibodies to the HA and Myc tags. WFS1^WT^ interacts with ATP1B1, as shown previously (column 5), and the p.E864K mutation does not affect this binding (column 6) (arrowhead: ATP1B1 band, arrow: WFS1 band). No co-immunoprecipitation was observed in control extracts co-transfected with either empty pCMV-Myc and pCMV-HA-Na^+^/K^+^ATPase β1 (column 1) or empty pCMV-HA and pCMV-Myc-WFS1^WT^ (column 2) or pCMV-Myc-WFS1^E864K^ (column 3). **B**, **C** Representative immunofluorescent labeling images of Myc-WFS1^WT^, Myc-WFS1^E864K^, and HA-ATP1B1 fusion proteins 72 h after transfection in MDCK cells. The subcellular localization shows that WFS1 (red) accumulates in the cytoplasm, most likely in the endoplasmic reticulum. ATP1B1 (green) is mostly accumulated at the plasma membrane, and in some cases forms aggregates in the cytoplasm. ATP1B1 tends to form more aggregates in the cytoplasm when co-transfected with WFS1^E864K^. Nucleus are highlighted with DAPI (blue) and the plasma membrane was stained with a Alexa-Fluor 647 Phalloidin (magenta). All images are projection of confocal optical section stack. Scale bar: 10 μm for all images. **D** Biotinylation of cell surface proteins was performed, on co-transfected HEK293T cells, to assess Na^+^/K^+^ATPase β1 subunit cell surface protein contents. WFS1^E864K^ mutant protein reduces cell surface localization of the Na^+^/K^+^ATPase β1 subunit, compared to WFS1^WT^ protein. Data are shown as mean ± SEM, percentage of control (*n* = 3, t-test, *p = 0.02).

We confirmed the previously reported interaction between WFS1 protein and the Na^+^/K^+^ATPase β1 subunit [17] using HEK293T cells transiently co-transfected with WFS1 (Myc-WFS1^WT^) and Na^+^/K^+^ATPase β1 subunit (HA-ATP1B1), a different mammalian cell type compared to the initial publication (Fig. 6A). Using anti-HA antibodies for immunoprecipitation, anti-HA and anti-Myc antibodies for immunoblotting, we detected a 35-50 KDa protein which corresponds to the Na^+^/K^+^ATPase β1 subunit and a ~100 KDa protein corresponding to WFS1. Na^+^/K^+^ATPase β1 subunit protein migrated as multiple bands, as initially observed, due to glycosylation differences [17]. In control extracts co-transfected with either empty pCMV-Myc and pCMV-HA-Na^+^/K^+^ATPase β1 or empty pCMV-HA and pCMV-Myc-WFS1^WT^ or pCMV-Myc-WFS1^E864K^, no co-immunoprecipitation was observed (Fig. 6A). The p.E864K mutation did not alter the interaction with the Na^+^/K^+^ATPase β1 subunit as the band corresponding to the mutant protein was observed by western blot on the immunoprecipitated fraction (Fig. 6A).

In MDCK7 cells, no overt difference in the level of expression nor localization of WFS1^E864K^ mutant protein was observed, in comparison to the wild-type WFS1 (green) (Fig. 6B). Both proteins aggregated in the cytoplasm, in the endoplasmic reticulum network. The Na^+^/K^+^ATPase β1 subunit localized mainly to the plasma membrane with some aggregates in the cytoplasm, co-localizing partially, in the cytoplasm, with both WFS1^WT^ and WFS1^E864K^ (Fig. 6B).

We then explored the impact of WFS1-Na^+^/K^+^ATPase β1 subunit interaction on the ATPase targeting to the cell surface. Previous study suggested that WFS1 was important for the maturation of the β1 subunit [17], a requirement for the α subunit transport to the plasma membrane [19, 39]. Using a biotinylation assay, we assessed the percentage of Na^+^/K^+^ATPase β1 subunit present at the cell surface compared to the whole Na^+^/K^+^ATPase β1 subunit protein fraction in transiently transfected HEK293T cells. When ATP1B1 is co-transfected with WFS1^E864K^, the amount of the ATPase subunit at the cell surface is significantly decreased compared to when it is co-transfected with the wild-type WFS1 (Fig. 6C). These results showed that the mutation altered the role of the Wolframin regarding Na^+^/K^+^ATPase β1 subunit.

## Discussion

Wolfram syndrome was first characterized in 1938 by Wolfram and Wagner [40]. Since then, technical advances have helped decipher the genetic underlying causes as well as better characterize the associated symptoms. *WFS1* was first identified as the causative gene of WS type I in 1998 [41], known to encompass more than 99% of the cases as of today [42]. Responsible for WS, variants of *WFS1* were also associated with different forms of inherited deafness and Wolfram-like syndrome [43]. Despite the emergence of multiple animal models, none of them present with an early onset hearing loss, hampering the understanding of WFS1 role in the auditory pathway [25–27, 44].

Here, we report a novel mouse model, the *Wfs1*^*E864K*^ line, harboring a missense mutation mimicking the human disease-causing p.E864K variant [4, 5, 30, 31]. Surprisingly, the homozygous *Wfs1*^*E864K*^ mutant mouse developed a severe vestibular deficit, starting at P21, associated with an abnormal morphology of the stereocilia bundles and a complete hair cell loss by P31. No alteration of the vestibular organs was observed on the unique WS affected individual temporal bone collected [10] and only a few cases of vestibular anomalies have been reported in humans so far, as vestibular assessment is performed only occasionally. For at least three studies of WS affected families, the vestibular dysfunction is alleged to stem from central defects [45–47]. However, in three other studies, different cases presented with vestibular areflexia and bilateral vestibular loss [48–50]. None of the affected individuals carrying the p.E864K variant had vestibular abnormalities, although this claim should be viewed with caution as no proper vestibular exploration was performed [4, 5, 30, 31]. One can hypothesize that WFS1 might be less indispensable in the human vestibular system compared to the murine one.

Both mutant models, *Wfs1*^*ΔExon8*^ and *Wfs1*^*E864K*^, displayed HL, although with different severity. The homozygous *Wfs1*^*ΔExon8*^ mutant that expresses nonfunctional Wolframin protein, have a late onset (at 4 months) mild to moderate progressive HL, while the homozygous *Wfs1*^*E864K*^ mice present with early onset (P23) progressive HL leading to profound HL by P29. In both models, all tested frequencies were affected.

Audiological characterization of the *Wfs1*^*ΔExon8*^ model resembled the one of the *Wfs1-KO* rat model, with late onset and slowly progressive hearing loss [24, 25]. However, all tested frequencies were affected for the mouse model while only low frequencies showed alterations in the Wfs1 deficient rat. Due to the late onset of the hearing impairment, it would be pertinent to evaluate or re-evaluate the audiology profile of the other animal models, such as *Wfs1*^*ΔExon2*^ mouse [28] or simple and double *wfs1* zebrafish lines [26, 27, 44]. Unfortunately, no straightforward comparison can be made with the human pathology as no clear phenotype/genotype correlation can be established in affected individuals. Genotypic as well as functional classifications have been made based on the type of variant and its location in the *WFS1* gene, predicting the effect on the gene and the protein. However, there is no direct link between the protein alteration/degradation and the phenotype, mostly due to the inter- and intra-familial disparities of the symptoms as well as the small size of the enrolled cohorts because of the rarity of the disease [51–53].

The absence of clear phenotype/genotype correlation goes for the p.E864K variant as well. The four studies reporting affected carriers of the disease-causing variant demonstrated a high variability in the severity, age of onset, and affected frequencies of the hearing impairment, as well as the associated symptoms [4, 5, 30, 31]. Contrary to other Wfs1 null models discussed previously (*Wfs1*^*ΔExon8*^, *Wfs1-KO* rat [25]), *Wfs1*^*E864K*^ have early onset (P23) and rapidly progressing hearing loss. By P31, the mutant mice are profoundly deaf for all tested frequencies.

To better characterize the importance of WFS1 in hearing, we intercrossed *Wfs1*^*ΔExon8*^ and *Wfs1*^*E864K*^ mice to generate *Wfs1*^*ΔExon8*/*E864K*^ mice. Surprisingly, although these mice are expressing only WFS1^E864K^, with no possible compensation from a WT protein, the mice did not develop any hearing deficit up to 12 months of age, the latest age tested (Figure S2). These data, taken with the absence of hearing deficit in heterozygous *Wfs1*^*E864K/+*^ and homozygous *Wfs1*^*ΔExon8*^ at early stages, suggested that WFS1^E864K^ protein is deleterious to the auditory pathway in a dose-dependent manner in the mouse. In addition, within a similar time frame than in human, only homozygous *Wfs1*^*E864K*^ mice develop hearing deficit, strengthening this hypothesis.

The hearing impairment measured in the *Wfs1*^*E864K*^ mice is associated with a complete OHC degeneration as early as P31 and an abnormal morphology of the IHC stereociliary bundles. Wfs1 is highly expressed in the SGN in both murine [11] and primate cochleae [12], so we analyzed wave I characteristics. Our study did not reveal significant reduction in wave I amplitude until P23, however, a complete disappearance of all waves was observed at P27 (Fig. 4). These results contradict the morphological preservation of IHCs and SGN in P31 mutant mice, thus suggesting that stria vascularis may account at least part of the mutant protein-induced hearing loss. Despite not being the primary cause of the hearing loss in this model, we cannot, however, exclude later stages SGN degeneration, as shown in other deafness mouse models (e.g., [54]).

While extremely severe, the OHCs degeneration might not be the primary cause of the hearing impairment observed in *Wfs1*^*E864K*^ mice but more likely a secondary effect of a stria vascularis alteration. The primary function of the stria is to maintain the ionic composition of the endolymph and generate the endocochlear potential, which is the electrochemical potential of the endolymph, the driving force for hair cell mechanoelectrical transduction. The loss of its integrity, with the extensive vacuolation of the intermediate cells, as seen in the *Wfs1*^*E864K*^ mice, certainly explain the decrease in EP and its OHCs-related dysfunction. While it is tempting to conclude from the absence of cell specific immunolabeling that both intermediate and marginal cells of the SV degenerated by P31, our semi-thin and TEM data still show the presence of different cell layers and no sign of nuclei degeneration at P27 and P31. However, the absence of key proteins such as NKCC1, KCNQ1, and Kir4.1 suggest that the presence of Wfs1 mutant protein leads eventually to an abnormal protein composition and thus a functional alteration of the marginal and intermediate cells of the stria vascularis. Marginal cells have proven to be important to maintain the ionic composition of the endolymph and consequently the endocochlear potential [55]. Changes in the composition of the endolymph may ultimately lead to a dysfunction of the mechanotransduction channel of the hair cells, resulting in deafness. To support this hypothesis, in addition to the stria vascularis cells, a large number of cell types, bathing in the endolymph or involved in the potassium recycling pathways within the scala media [56], seems to present some degree of vacuolization, namely inner and outer sulcus cells, interdental cells and Reissner’s membrane cells.

Moreover, EP is thought to be generated by the unidirectional transport of K^+^ from the stria vascularis to the endolymph [57], through ion channels and transporters in the marginal and intermediate cells of the stria [58, 59]. This is corroborated by the study of other mouse models of key proteins of the stria vascularis. Null mutations in either *Kcnq1* [60], *Kcne1* [61], or *Kcnj10* [62] are associated with a collapse of the Reissner’s membrane, a severe atrophy of the stria vascularis, and a degeneration of both the organ of Corti and SGN, at early stages. All these mutants present with a disruption of the endolymph production and K^+^ homeostasis in the inner ear, leading to both deafness and vestibular dysfunction. All these genes encode proteins that are expressed in the marginal cells of the stria vascularis, such as KCNQ1 (encoded by *Kcnq1* gene) and KCNE1 (*Kcne1*) [63], or in the apical membrane of the intermediate cells, such as Kir4.1(*Kcnj10*) [64]. They are involved in the ionic composition of the endolymph, as is the Na^+^/K^+^ ATPase [65]. This cation pump, composed of one α and one β subunit and one FXYD protein [66], is broadly expressed in mammal cells with α and β specific isoforms composition depending on the cell type [18]. The β1 subunit, a known partner of WFS1 [17], is predominantly expressed, along with the α1 subunit [22, 23], in the marginal cells of the murine stria vascularis, where it is thought to be responsible for the folding and transport of the α subunit [39]. In the murine cochlear wall, immunoprecipitation assays have shown that, even though other subunits are present and can interact, the α1-β1 heterodimer is predominant over others [67]. Interestingly, Wolframin shares a similar expression pattern in the stria vascularis [11], enabling their interaction in the marginal cells at early stages. To note, a new RNAseq analysis, specific to the adult (P30) mouse lateral wall, showed that while both *Wfs1* and *ATP1B1* were still expressed concomitantly, they were predominantly expressed in the intermediate cells, and in, a lesser extent, in the marginal cells [68]. In this study, we showed that the disease-causing variant p.E864K did not affect the interaction between the two partners, however, it affected the targeting of the Na^+^/K^+^ ATPase β1 subunit to the cell surface, potentially prohibiting the Na^+^/K^+^ pump proper function. Based on previous study, the Na^+^/K^+^ ATPase β1 subunit might be retained in the ER, where the interaction with WFS1 is thought to take place [17]. Moreover, Na^+^/K^+^ ATPase subunits are able to exit the ER only if they are properly assembled as a Na^+^/K^+^ ATPase α:β-complex, any subunits with assembly or folding alterations being retained in the ER and degraded [69]. WFS1 binds the Na^+^/K^+^ ATPase β1 subunit while unfolded, therefore one can hypothesize that the disease-causing protein might prevent the folding of the β1 subunit. It would then prevent the formation of the α1:β1-complex and lead to its subsequent degradation, decreasing the level of Na^+^/K^+^ ATPase β1 subunit and functional Na^+^/K^+^ ATPase pump at the plasma membrane. The dilated rough ER observed at P27 in the marginal cells of the stria vascularis of the mutant mice tends to confirm this hypothesis.

In addition, the Na^+^/K^+^ ATPase is also critical in the formation of the vestibular endolymph which would explain the unexpected vestibular phenotype observed in the *Wfs1*^*E864K*^ mutant mice [70].

Another hypothesis that could explain the phenotype of the mutant mice is a decrease of the ATP concentration in the inner ear cells. The commonly accepted role of Wolframin is its involvement in ER stress and Ca^2+^ homeostasis, and more particularly at the mitochondria-associated ER membranes (MAMs), where lack of WFS1 is associated with an impairment of ER to mitochondria Ca^2+^ transfer ultimately leading to a mitochondrial dysfunction and apoptosis [13]. The p.E864K mutant might impact this pathway, resulting in a decrease of the ATP concentration in the cell, due to dysfunctional mitochondria. This decrease, combined with less functional Na^+^/K^+^ ATPase at the cell surface, would drastically impact the intracellular as well as the endolymphatic K^+^ ion levels and consequently the endocochlear potential [58]. While this hypothesis would require additional investigations, it could explain the stria vascularis collapse as well as the subsequent HC degeneration observed in the *Wfs1*^*E864K*^ mutant mice.

In conclusion, we report a novel mouse model for Wolfram syndrome, *Wfs1*^*E864K*^, with severe hearing and vestibular deficits. The hearing loss is associated with a complete loss of outer hair cells and fused stereocilia of the inner hair cells of the organ of Corti, most likely a consequence of the extreme vacuolization of the intermediate cells of the stria vascularis. We showed that the mutant protein still binds to the Na^+^/K^+^ATPase β1 subunit and affects its localization to the cell surface, presumably by retaining it in the ER, preventing a normal function of the Na^+^/K^+^ATPase pump. In the context of the stria vascularis, it would translate into an abnormal ionic homeostasis, leading eventually to a severe alteration of the stria vascularis itself and more broadly the organ of Corti epithelium. Our data strongly suggest that WFS1 plays a crucial role in the auditory pathway, as a key element in the maintenance of normal endocochlear potential and ionic transfer.

## Supplementary information

Supplemental Material

Checklist

## Data Availability

All data needed to evaluate the conclusions of the paper are present in the paper and/or the Supplementary Materials.
