# Supplementary material for: Wfs1E864K knock-in mice illuminate the fundamental role of Wfs1 in endocochlear potential production
Source: Cell Death Dis. 2023 Jun 29;14(6):387. doi: 10.1038/s41419-023-05912-y (PMC10310813; doi:10.1038/s41419-023-05912-y)
Supplement: Supplementary file 1 — Supplemental Material [file 41419_2023_5912_MOESM1_ESM.pdf]

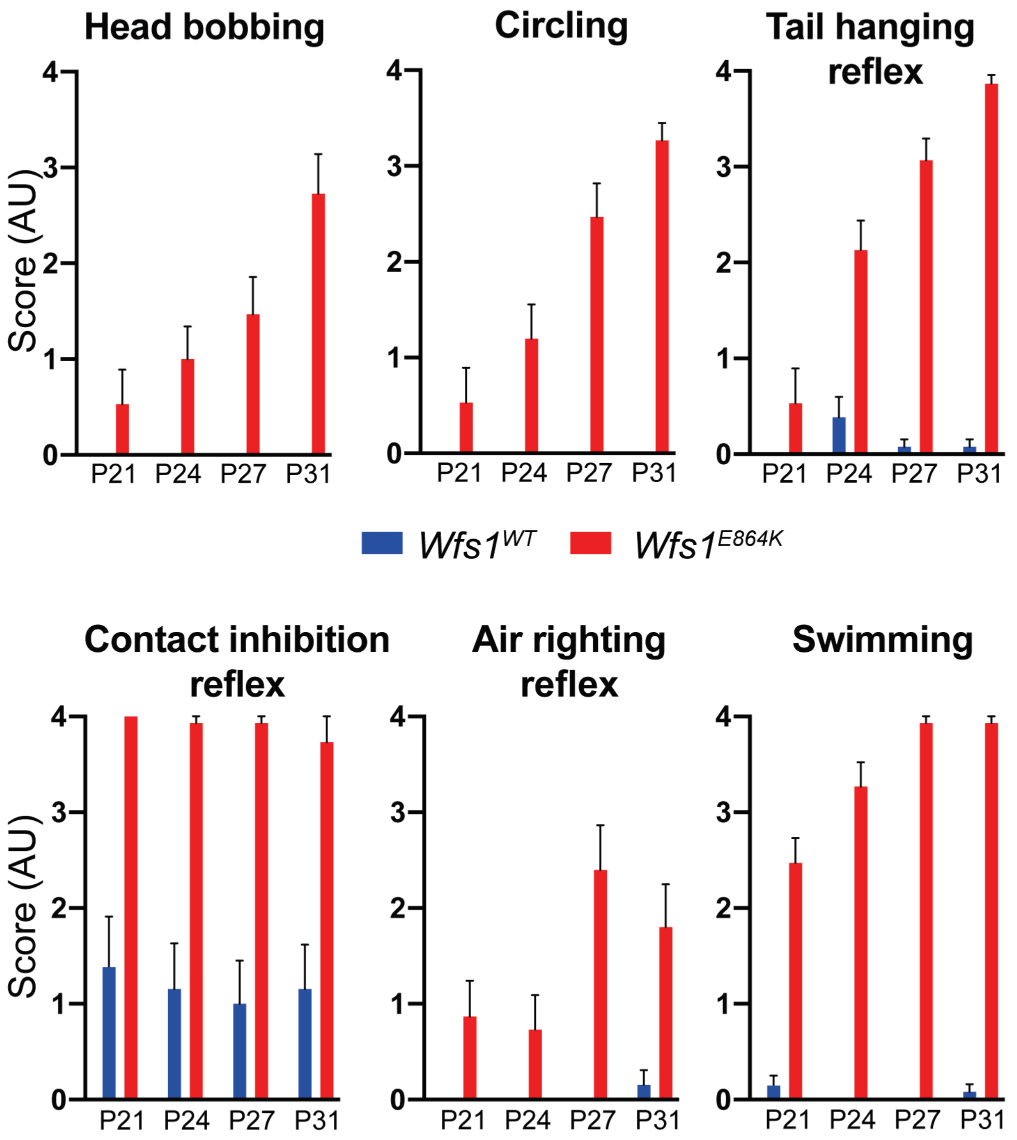

Supplemental Figure 1. *Wfs1^{E864K}* mice develop severe vestibular deficits. Evaluation of vestibular deficits in P21, P24, P27 and P31 *Wfs1^{WT}* (blue; n = 15) and *Wfs1^{E864K}* (red; n = 13) mice. Six different tests were evaluated, scored from 0 to 4 in arbitrary units (AU). *Wfs1^{E864K}* mice exhibited strong, progressing, vestibular deficits, statistically different from vestibular behavior of their WT littermates (t-test, $p<0.001$ for all tests and all time points). Data are mean ± SEM.

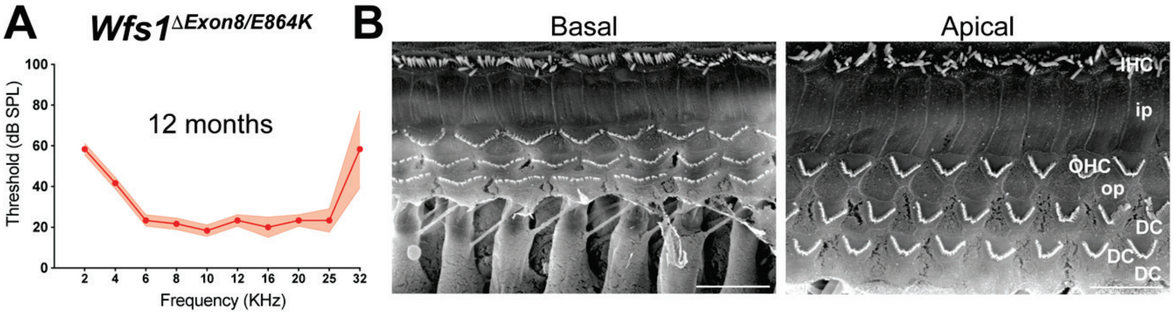

Supplemental Figure 2. ***Wfs1***$^{\Delta Exon8/E864K}$ **mouse ABR and hair cell morphology are normal at 12 months.** We intercrossed *Wfs1*$^{\Delta Exon8}$ and *Wfs1*^{E864K} mice to generate heterozygote mutants and assess a potential hearing loss. (A) Averaged ABR thresholds of *Wfs1*$^{\Delta Exon8/E864K}$ mice at 12 months in response to 2, 4, 6, 8, 10, 12, 16, 20, 25 and 32 KHz tone-bursts, with sound pressure level (SPL) of 0 to 100 dB. The *Wfs1*$^{\Delta Exon8/E864K}$ mice showed classical ABR threshold ranges, with no hearing alteration. (B) Scanning electron micrographs of the organ of Corti from the apical and basal regions of *Wfs1*$^{\Delta Exon8/E864K}$ mice at 12 months with intact outer and inner hair cells and no overt morphological alteration. Scale bar: 15 μm.

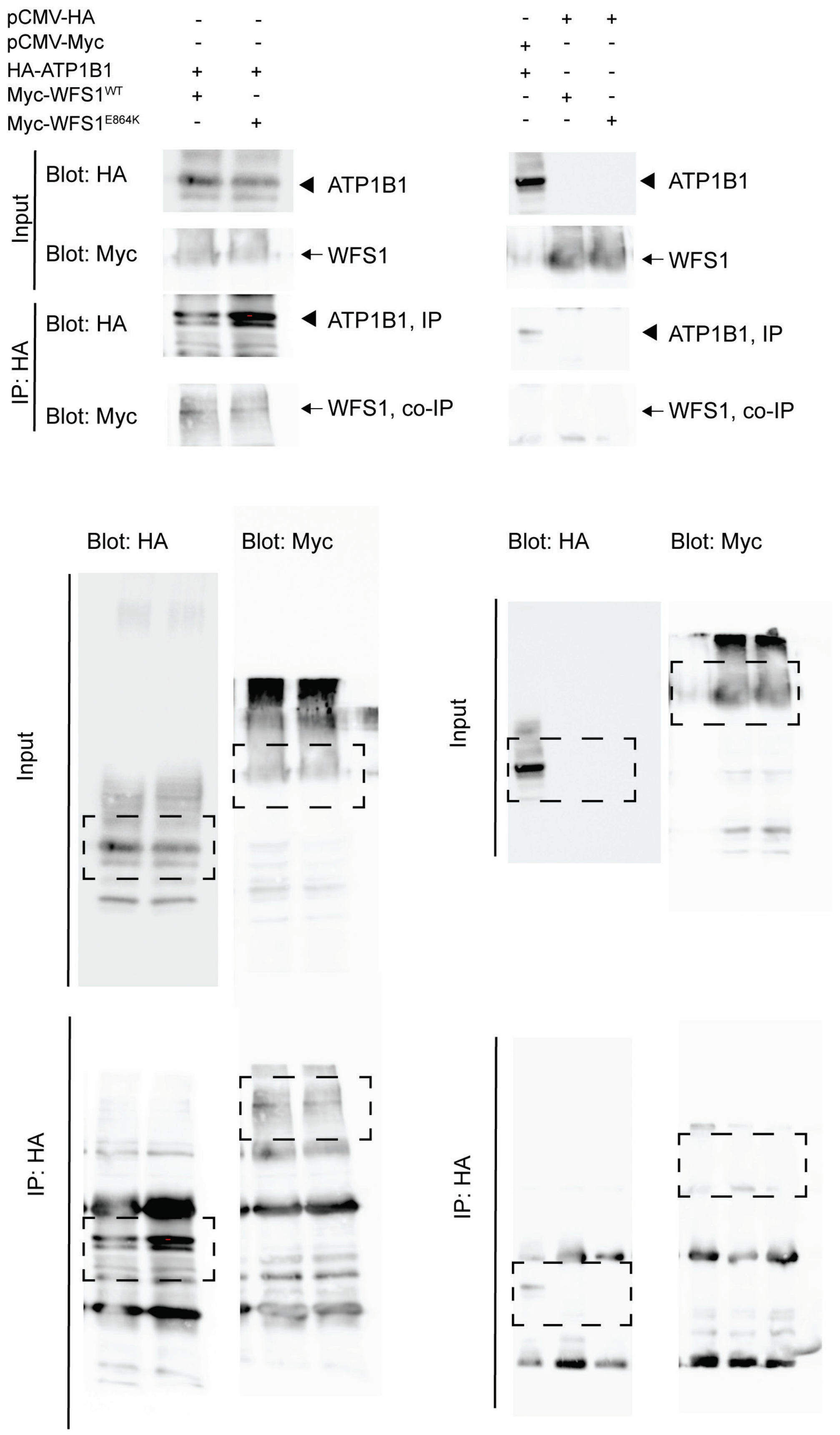

Supplemental Figure S3. Uncropped immunoblots used in Figure 6.

Table S1. Two-way ANOVA statistical values for Figure 1A and 1B.

Age	Factor	F (DFn, DFd)	P value
4-month-old	Genotype	F (1, 180) = 64,3	<0,0001
	Frequency	F (9, 180) = 111,2	<0,0001
	Interaction	F (9, 180) = 0,7001	0,7082
10-month-old	Genotype	F (1, 140) = 205,6	<0,0001
	Frequency	F (9, 140) = 89,46	<0,0001
	Interaction	F (9, 140) = 1,039	0,4124

Table S2. Two-way ANOVA statistical values for Figure 2D and 2E.

Figure	Factor	F (DFn, DFd)	P value
Figure 2D	Genotype	F (1, 81) = 111,2	<0,0001
	Day	F (2, 81) = 1,347	0,2659
	Interaction	F (2, 81) = 0,4889	0,6151
Figure 2E	Genotype	F (1, 104) = 408,2	<0,0001
	Day	F (3, 104) = 12,69	<0,0001
	Interaction	F (3, 104) = 14,12	<0,0001

Table S3. Two-way ANOVA statistical values for Figure 3A.

Age	Factor	F (DFn, DFd)	P value
P21	Genotype	F (2, 220) = 2,256	0,1072
	Frequency	F (9, 220) = 69,3	<0,0001
	Interaction	F (18, 220) = 0,5483	0,9319
P23	Genotype	F (2, 220) = 4,379	0,0136
	Frequency	F (9, 220) = 16,09	<0,0001
	Interaction	F (18, 220) = 0,37	0,9917
P25	Genotype	F (2, 220) = 128,1	<0,0001
	Frequency	F (9, 220) = 56,35	<0,0001
	Interaction	F (18, 220) = 5,565	<0,0001
P27	Genotype	F (2, 220) = 7526	<0,0001
	Frequency	F (9, 220) = 107,1	<0,0001
	Interaction	F (18, 220) = 27,10	<0,0001
P29	Genotype	F (2, 220) = 12386	<0,0001
	Frequency	F (9, 220) = 171,4	<0,0001
	Interaction	F (18, 220) = 44,51	<0,0001
P31	Genotype	F (2, 220) = 8484	<0,0001
	Frequency	F (9, 220) = 116,2	<0,0001
	Interaction	F (18, 220) = 31,01	<0,0001
